# Editorial: Insights in gastroenterology: 2022

**DOI:** 10.3389/fmed.2024.1367555

**Published:** 2024-02-06

**Authors:** Angel Lanas, Gonzalo Hijos-Mallada

**Affiliations:** ^1^Department of Medicine, University of Zaragoza, Zaragoza, Spain; ^2^Biomedical Research Networking Center in Hepatic and Digestive Diseases (CIBERehd), Madrid, Spain; ^3^Aragon Health Research Institute (IIS Aragon), Zaragoza, Spain; ^4^Service of Digestive Diseases, University Clinic Hospital Lozano Blesa, Zaragoza, Spain

**Keywords:** gastrointestinal submucosal tumors, digestive endoscopy, pancreatic cancer, colorectal cancer, fibrinogen, inflammatory bowel disease, diet

*Insights in gastroenterology: 2022* was a Research Topic designed to highlight the latest advancements in the growing field of gastroenterology. Gastroenterology research is increasing in complexity, encompassing a diverse range of sub-disciplines, as illustrated by the contributing articles within this Research Topic.

Gastrointestinal endoscopy is one of these areas undergoing major developments, with several emerging endoscopic techniques in recent years. Submucosal tunnel endoscopic resection (STER) is an advanced procedure, developed for the resection of gastrointestinal submucosal tumors (SMTs). STER offers the advantages of maintaining mucosal integrity, given that it is less invasive than surgery (1, 2). However, scarce evidence is available regarding risk factors associated with technical complexity and complications. To shed light on this topic, Lv et al. performed a retrospective analysis of the outcomes of 66 SMTs located in the upper gastrointestinal tract managed by STER. The median size of the SMTs was 18 mm, and most (80%) were originated in the muscularis propria. Complete resection and en bloc resection were achieved in 66 (100%) and 64 (97%) patients, with a median procedure time of 45 min. STER-related complications were observed in 10 (15%) of the patients, all managed by conservative treatment. Multivariable analysis revealed that tumor size was an independent risk factor for prolonged procedure time (>60 min), complications, and long hospital stay (>6 days). A tumor size of ≥25 mm was identified as the optimal threshold to define high-risk patients. In such cases, STER should be performed by a more experienced team, or other treatments should be considered.

Beyond the emerging endoscopic techniques, image and video technologies are a cornerstone for the ongoing evolution of endoscopy. Chen et al. carried out a comprehensive review of endoscopic technologies across various applications, emphasizing the critical importance of image quality for accurate diagnosis. In this line, 4K ultra-high definition is gaining significance in complex procedures, albeit with associated higher costs and hardware demands. Capsule endoscopy allows examination of hard-to-reach gastrointestinal areas, offering varying fields of view, with some devices provided with a 360° panoramic lateral viewing. Pediatric endoscopes are smaller (3–6 mm diameter) and may provide a wide field of view (130–170°), akin to ultrathin scopes (5–6 mm, 120–150°). Advances in image technology have enabled these devices to deliver resolutions and depth perception comparable to standard endoscopes. Furthermore, some incorporate advanced imaging techniques (NBI, FICE, or i-scan). Besides their utility in pediatric patients or narrow areas, these endoscopes can be highly useful in endoscopic-guided feeding tube placement. This latter indication, along with other procedures requiring precision, could benefit from innovations in movement control for endoscopy. These include magnetic-assisted capsule endoscopy or robotic-assisted endoscopy. In addition to these developments, ongoing advancements such as the integration of artificial intelligence, optical coherence tomography, confocal laser endomicroscopy, and 3D imaging have the potential to further revolutionize gastrointestinal endoscopy.

Gastrointestinal oncology is another sub-discipline that has seen remarkable research activity in recent years. Pancreatic cancer (PC) incidence is increasing worldwide (3). However, survival rates have barely changed, so it remains one of the most lethal cancers (4, 5). To evaluate the primary factors influencing PC prognosis and estimate current and future survival trends, Li et al. conducted a period analysis to determine the 5-year relative survival rate (RSR) of PC patients from 2002 to 2016. Clinical information was extracted from the SEER database of the National Cancer Institute (US population), comprising 39,700 PC patients. The study revealed an increase in the 5-year RSR from 7.9% in 2002 to 23.7% in 2016. A generalized linear model, applied to predict the RSR of PC patients diagnosed during 2017–2021, estimated a further increase to 33.9%. Therefore, the RSR of PC patients is expected to remain significantly low. Older age (>75 years), high differentiation grade, and the presence of distant metastases at diagnosis were factors consistently associated with lower RSR. These findings underscore the critical need for the development of new strategies for early PC detection. Regarding early oncologic diagnosis, colorectal cancer (CRC) stands out as one of the pathologies with more validated screening strategies. These include fecal immunochemical tests (6), multitarget stool DNA tests (7), or blood-based tests such as blood-cell-based markers (8). In this context, Wang et al. explored the potential relationship between serum fibrinogen and the primary precursor of CRC, advanced colorectal adenoma. This association was evaluated in a case-control study conducted on hospitalized patients, including 566 individuals diagnosed with advanced adenomas and 3172 healthy controls (normal colonoscopies). A linear correlation was observed between serum fibrinogen and advance adenoma, with the higher incidence of advanced adenoma found in patients with elevated fibrinogen levels (quartiles 3–4). This finding suggests that fibrinogen may play a role in adenoma–carcinoma progression and could serve as a potential biomarker for CRC screening.

Finally, the intricate interplay between diet, microbiota, and gastrointestinal inflammation remains an ongoing focus of research. Inflammatory bowel disease (IBD) has a complex pathogenesis, with these interactions playing an essential role (9). In translational research, Abcb1a knockout (KO) mice serve as a valuable IBD model (10). In an experiment conducted by Stensballe et al., 31 KO mice and 30 wild-type (WT) specimens were randomly allocated to receive diets supplemented with either casein or red meat. The primary objective was to elucidate potential interactions between diet, the gut microbiome, and the immune system. Histologic examinations revealed that WT groups exhibited normal colon histology, whereas KO mice displayed a colitis phenotype, with no significant differences between diets. The authors conducted a thorough quantitative proteomic inflammation profiling of gastrointestinal tissue and urine, revealing significant changes in the colon proteome of KO mice, allowing for differentiation between KO and WT mice. A similar trend was observed in the ileum and urine proteomes, although the variation was not sufficient to discriminate WT from KO mice. Elevated levels of neutrophil extracellular trap (NET) components were found in the colon, ileum, and urine. Likewise, gut microbiota profiles of WT and KO mice were significantly different, while only minor distinctions were detected in relation to the intake of red meat. These findings have the potential to enhance our understanding of IBD pathogenesis and may serve as a foundation for exploring new translational IBD biomarkers based on NETs.

## Author contributions

AL: Writing—review & editing. GH-M: Writing—original draft.

## References

[1] Lv XH Wang CH Xie Y. Efficacy and safety of submucosal tunneling endoscopic resection for upper gastrointestinal submucosal tumors: a systematic review and meta-analysis. Surg Endosc. (2017) 31:49–63. 10.1007/s00464-016-4978-7 27287907

[2] Chen T Lin ZW Zhang YQ Chen WF Zhong YS Wang Q. Submucosal tunneling endoscopic resection vs. thoracoscopic enucleation for large submucosal tumors in the esophagus and the esophagogastric junction. J Am Coll Surg. (2017) 225:806–16. 10.1016/j.jamcollsurg.2017.09.002 28923691

[3] Khalaf N El-Serag HB Abrams HR Thrift AP. Burden of pancreatic cancer: from epidemiology to practice. Clin Gastroenterol Hepatol. (2021) 19:876–84. 10.1016/j.cgh.2020.02.054 32147593 PMC8559554

[4] Sung H Ferlay J Siegel RL Laversanne M Soerjomataram I Jemal A. Global cancer statistics 2020: GLOBOCAN estimates of incidence and mortality worldwide for 36 cancers in 185 countries. CA Cancer J Clin. (2021) 71:209–49. 10.3322/caac.21660 33538338

[5] Klein AP. Pancreatic cancer epidemiology: understanding the role of lifestyle and inherited risk factors. Nat Rev Gastroenterol Hepatol. (2021) 18:493–502. 10.1038/s41575-021-00457-x 34002083 PMC9265847

[6] Navarro M Hijos G Ramirez T Omella I Carrera-Lasfuentes P Lanas Á. Fecal hemoglobin concentration, a good predictor of risk of advanced colorectal neoplasia in symptomatic and asymptomatic patients. Front Med. (2019) 6:91. 10.3389/fmed.2019.00091 31131279 PMC6510055

[7] Shaukat A Levin TR. Current and future colorectal cancer screening strategies. Nat Rev Gastroenterol Hepatol. (2022) 19:521–31. 10.1038/s41575-022-00612-y 35505243 PMC9063618

[8] Hernandez-Ainsa M Velamazan R Lanas A Carrera-Lasfuentes P Piazuelo E. Blood-cell-based inflammatory markers as a useful tool for early diagnosis in colorectal cancer. Frontiers in medicine. (2022) 9:843074. 10.3389/fmed.2022.843074 35795635 PMC9252519

[9] Imhann F Vich Vila A Bonder MJ Fu J Gevers D Visschedijk MC. Interplay of host genetics and gut microbiota underlying the onset and clinical presentation of inflammatory bowel disease. Gut. (2018) 67:108–19. 10.1136/gutjnl-2016-312135 27802154 PMC5699972

[10] Panwala CM Jones JC Viney JL A. novel model of inflammatory bowel disease: mice deficient for the multiple drug resistance gene, mdr1a, spontaneously develop colitis. J Immunol. (1998) 161:5733–44. 10.4049/jimmunol.161.10.5733 9820555

